# Supplementary material for: Stability analysis of chaotic systems from data
Source: Nonlinear Dyn. 2023 Feb 10;111(9):8799–819. doi: 10.1007/s11071-023-08285-1 (PMC10076397; doi:10.1007/s11071-023-08285-1)
Supplement: Supplementary file 1 — (pdf 5173 KB) [file 11071_2023_8285_MOESM1_ESM.pdf]

Supplementary Material for Stability analysis of chaotic systems from data

Georgios Margazoglou[1] and Luca Magri[1,2]

[1)] *Imperial College London, Aeronautics Department, South Kensington Campus London SW7 2AZ, United Kingdom*

[2)] *The Alan Turing Institute, 96 Euston Road, NW1 2DB, London, United Kingdom*

(*Electronic mail: l.magri@imperial.ac.uk)

(*Electronic mail: g.margazoglou@imperial.ac.uk)

(Dated: 11 October 2022)

Here we provide additional results and figures that extend the findings of the main paper.

I. LORENZ 63

We firstly start with additional results of Lorenz 63. In Fig. 1 (a-c) we plot the Probability Density Functions (PDF) of the three finite-time Lyapunov exponents (FTLEs). Then in Fig. 1 (d-f) we plot the PDFs of the sums of FTLEs, Λ_1, $\Lambda_1+\Lambda_2$, and $\Lambda_1+\Lambda_2+\Lambda_3$.

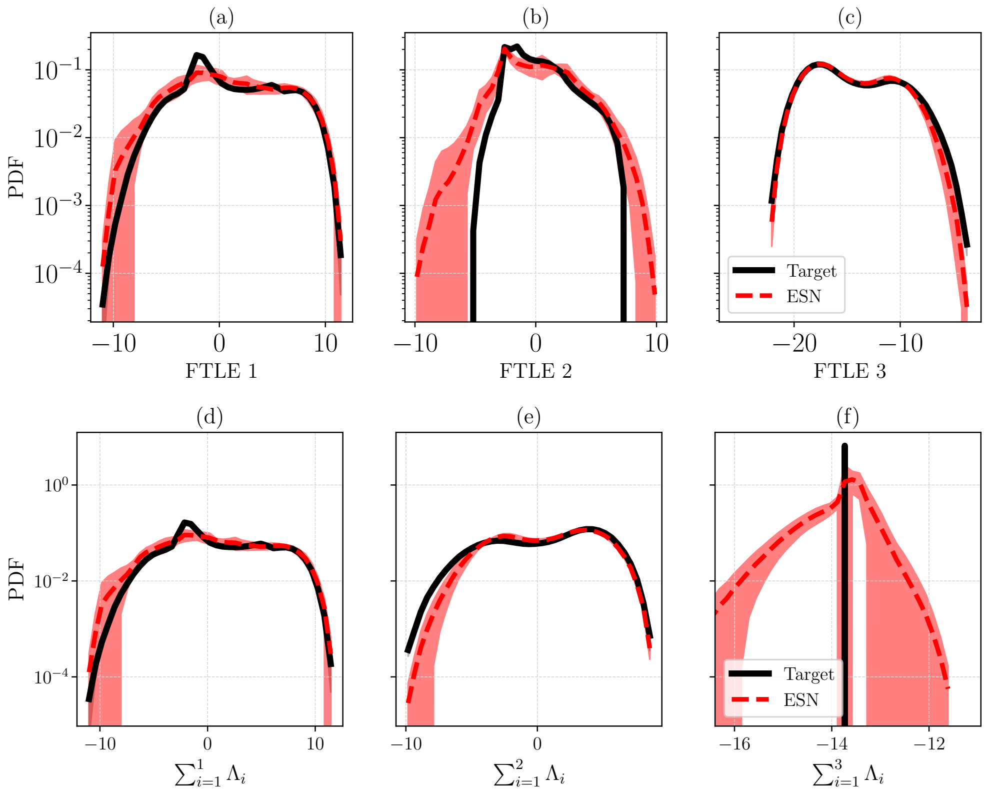

FIG. 1. (a-c) PDFs of individual FTLEs, (d-f) PDFs of sums of FTLEs. In alls cases the y axis is logarithmic. Here the case with $\sum_{i=1}^{6}\Lambda_i(t)=\text{const}$ for target, which is not satisfied by the ESN. Red line is for ESN and black line for Target.

In Fig. 2 we show at the first two columns of plots the distribution of the finite-time covariant Lyapunov exponents (FTCLEs) Λ_i^c, $i=1,2,3$ on the topology of the attractor. This provides information on the finite-time growth or decay of the corresponding CLVs at each point of the attractor. High positive values indicate reduced predictability horizon. Then, at the two last columns of plots of Fig. 2 we show the sums of FTLEs Λ_1, $\Lambda_1+\Lambda_2$, and $\Lambda_1+\Lambda_2+\Lambda_3$. These quantities indicate how 1-,2-,3-dimensional volumes grow or decay at finite-time intervals on the topology of the attractor. The total volume contraction given by $\Lambda_1+\Lambda_2+\Lambda_3$ is a negative constant in the target system, which is marginally not achieved by the ESN. The ESN is still peaked around the

same negative value; see Fig. 1(f).

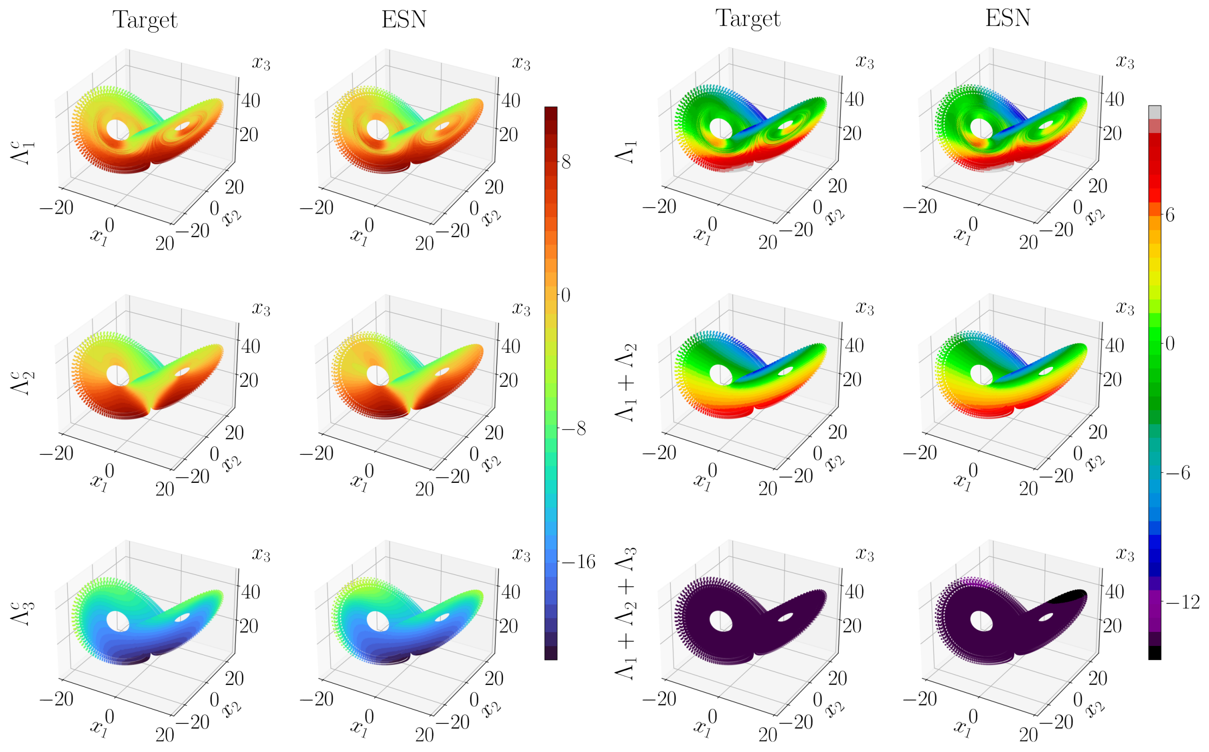

FIG. 2. The two first columns of plots are results for finite-time covariant Lyapunov vectors Λ_i^c. The two last columns are results for the summed finite-time Lyapunov exponents Λ_i. In each group the left column are the target results and the right column the ESN results for a $300\tau_\lambda$ trajectory of the Lorenz 63 attractor. The colouring in the first group is by FTCLEs: Λ_1^c, Λ_2^c, and Λ_3^c. The colouring in the second group is by sums of the FTLEs: Λ_1, $\Lambda_1 + \Lambda_2$, and $\Lambda_1 + \Lambda_2 + \Lambda_3$. Of course $\Lambda_1^c \equiv \Lambda_1$ by definition.

II. RÖSSLER

Here we present additional results for the Rössler attractor. In Fig. 3 (a-c) we plot the PDFs of the three FTLEs. Then in Fig. 3 (d-f) we plot the PDFs of the sums of FTLEs, Λ_1, $\Lambda_1 + \Lambda_2$, and $\Lambda_1 + \Lambda_2 + \Lambda_3$.

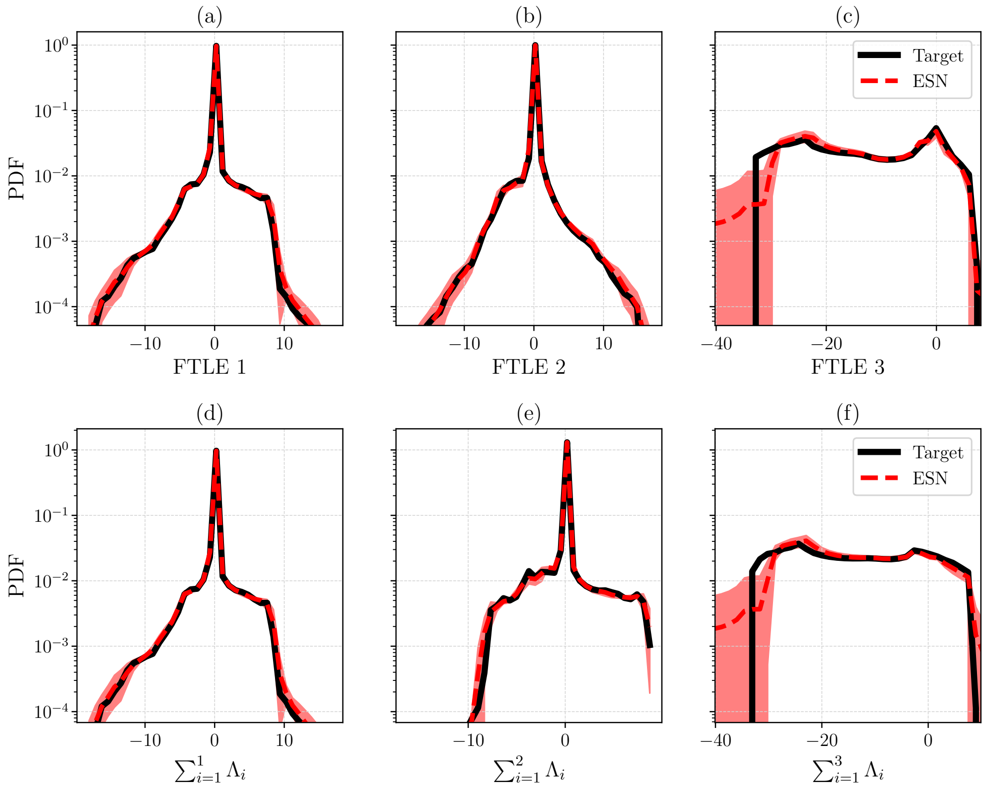

FIG. 3. (a-c) PDFs of individual FTLEs, (d-f) PDFs of sums of FTLEs. In alls cases the y axis is logarithmic. Red line is for ESN and black line for Target.

Then in Fig. 4 we compare the distribution of the angles between the three CLVs (the first two columns of plots) on the topology of the attractor. Accordingly the last two columns of plots are the sums of FTLEs on the topology of the attractor. The agreement is good in all cases, with some small deviations in some parts of the attractor in the case of the $\theta_{U,S}$ and $\theta_{N,S}$.

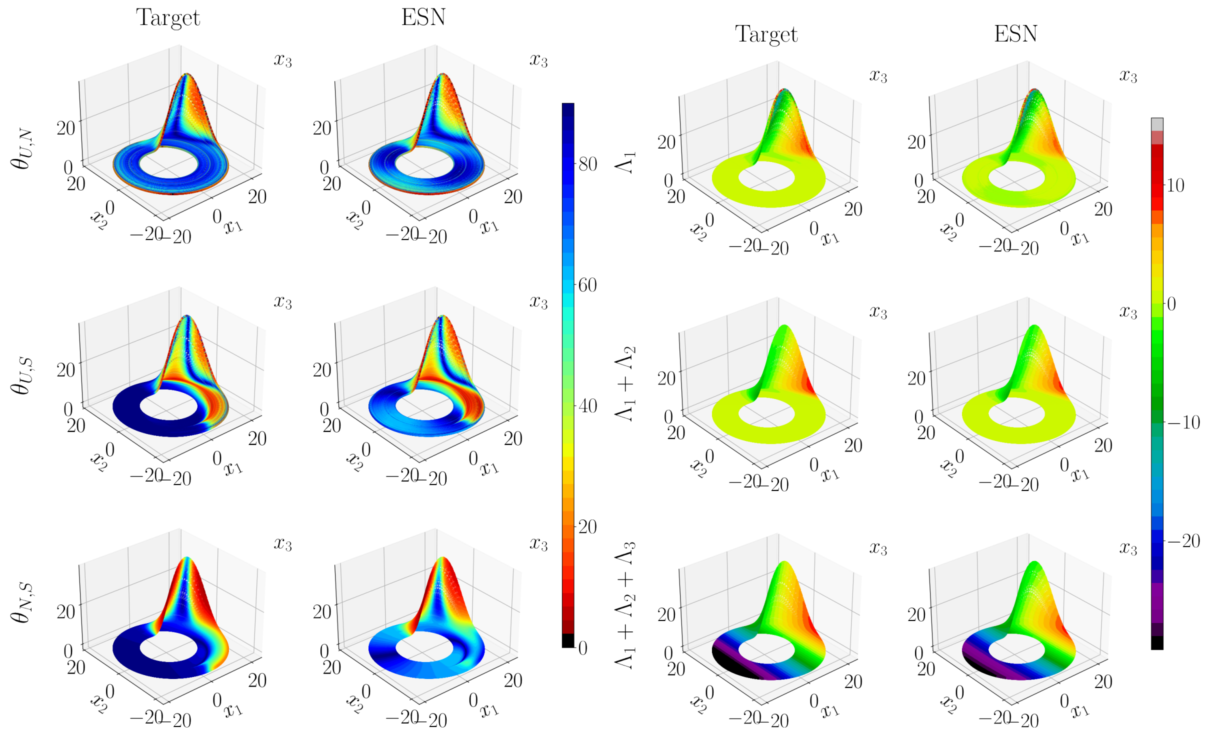

FIG. 4. The two first columns of plots are results for angles between CLVs. The two last columns are results for the summed finite-time Lyapunov exponents Λ_i. In each group the left column are the target results and the right column the ESN results for a $300\tau_\lambda$ trajectory of the Rössler attractor. The colouring in the first group is by angles between the CLVS: $\theta_{U,N}$, $\theta_{U,S}$, and $\theta_{N,S}$. The colouring in the second group is by sums of the FTLEs: Λ_1, $\Lambda_1 + \Lambda_2$, and $\Lambda_1 + \Lambda_2 + \Lambda_3$.

III. CHARNEY-DEVORE

Here we present additional results for the Charney-DeVore system. In Fig. 5 the results are related to the statistics of FTLEs, comparing between the ESN (red) and the target (black). The agreement in most cases is good. Only for $\sum_{i=1}^{6} \Lambda_i(t)$ which is a constant for the target system, the ESN gets a slightly different behavior, which is still peaked close to the value of the target.

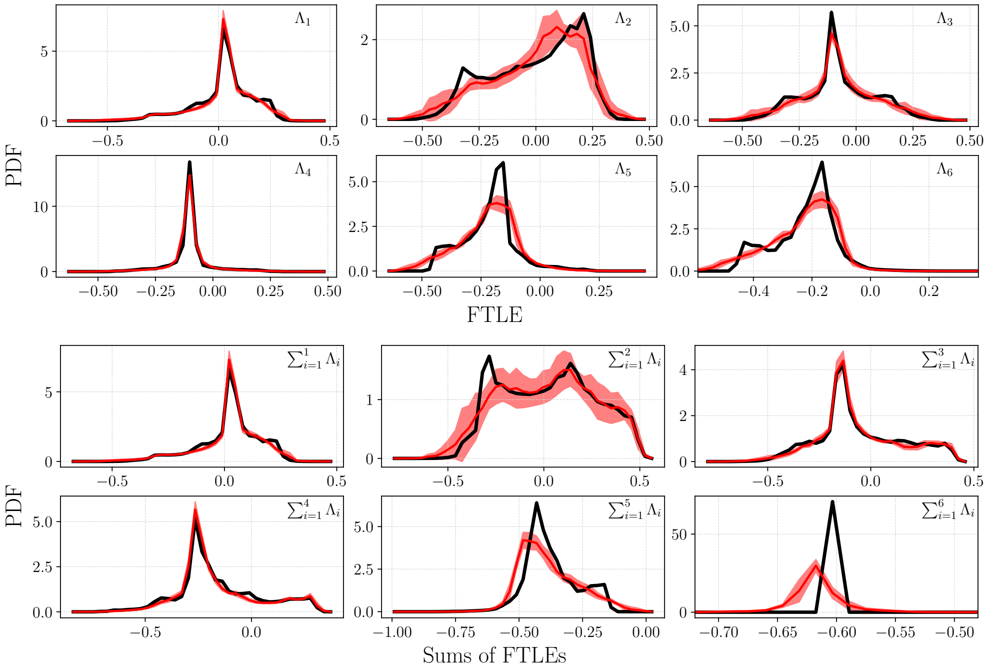

FIG. 5. Top six plots are individual FTLEs, and bottom six plots are the sums of FTLEs. In all cases the y axis is linear. Here the case with $\sum_{i=1}^{6} \Lambda_i(t) = \text{const}$ for target, which is not satisfied by the ESN. Red line is for ESN and black line for Target.

As a more qualitative comparison, in the example of CdV, we demonstrate in Fig. 6(a) the distribution of the CLV angles between the three subspaces, and in 6(b) the distribution of the FTCLEs in a truncated physical space composed only of the 3 first variables, x_1, x_2, x_3, for purely visual purposes. In Fig. 6(c) we do the same for sums of FTLEs.

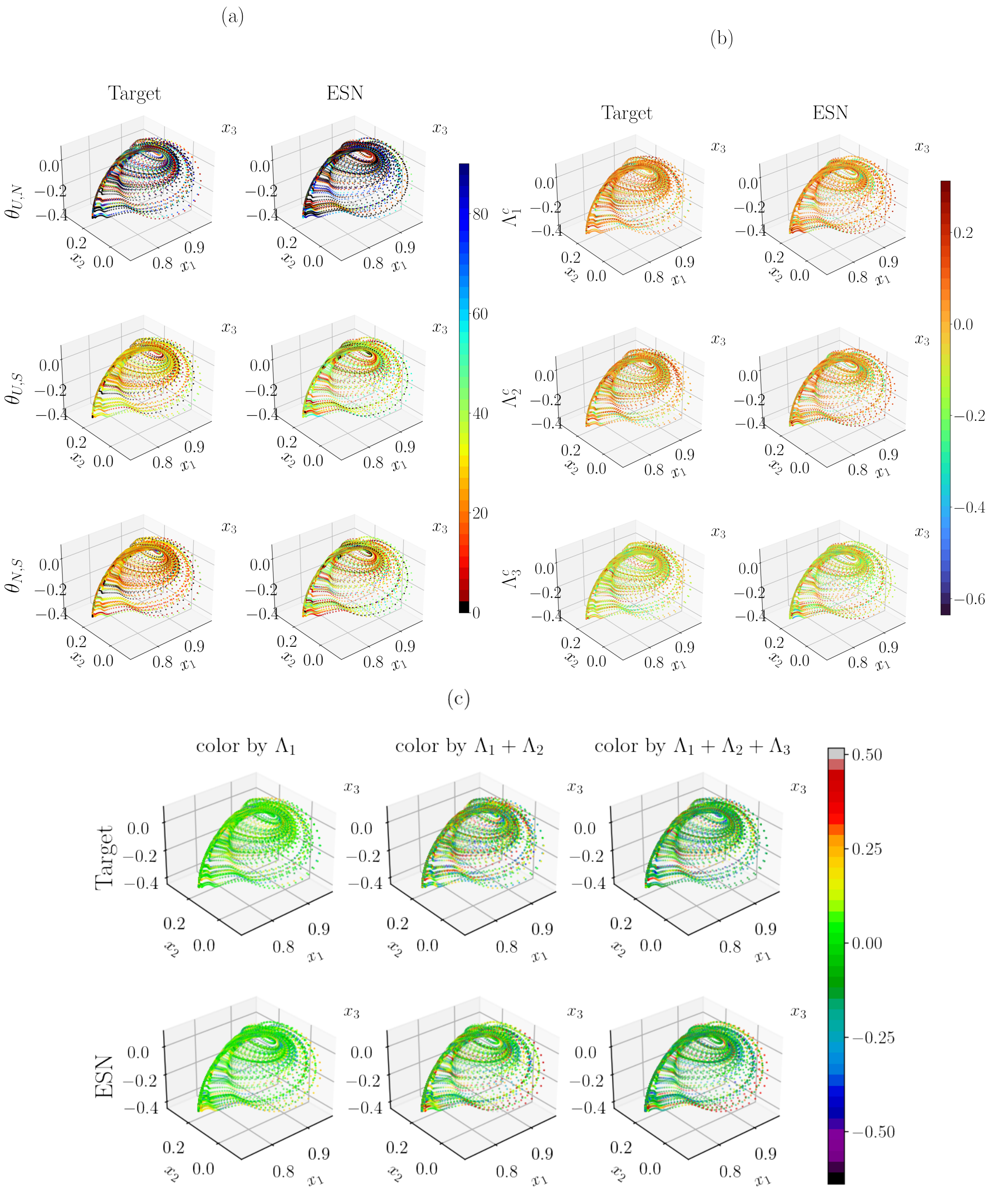

FIG. 6. Comparison of Target and ESN for a $150\tau_\lambda$ trajectory of the Charney-DeVore system in the test set, coloured by (a) the three instantaneous angles between the manifolds composed by CLVs, (b) by the three first FTCLEs Λ^c, and (c) sums of the first three FTLEs Λ.

IV. LORENZ 96

Here we present additional results for the Lorenz 96 system at $D = 20$ degrees of freedom and for $F = 8$. In Fig. 7 the results are related to the statistics of FTLEs, comparing between the ESN (red) and the target (black). The agreement in most cases is good. Only for $\sum_{i=1}^{20} \Lambda_i(t)$ which is a constant for the target system, the ESN gets a slightly different behavior, which is still peaked close to the value of the target.

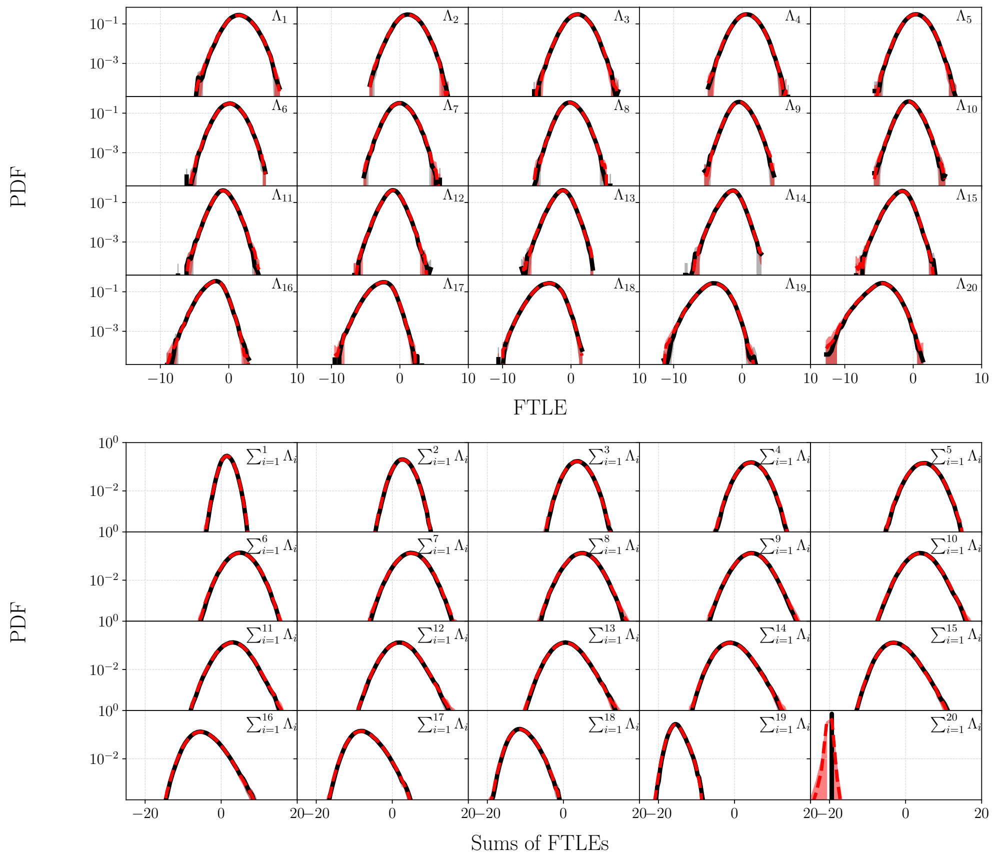

FIG. 7. Top 20 plots are individual FTLEs, and bottom 20 plots are the sums of FTLEs. In all cases the y axis is logarithmic. Red dashed line is for ESN and black line for Target. Each group of plots shares the same x and y range.
